# Depressive symptoms and cognitive function as independent correlates of daily functioning in older adults with cognitive complaints

**DOI:** 10.1192/j.eurpsy.2026.11561

**Published:** 2026-07-31

**Authors:** S. Yoon, S. Y. Kim

**Affiliations:** 1 Psychiatry, Daegu Catholic University School of Medicine; 2 Psychiatry, Daegu Catholic University Medical Center, Daegu, Korea, Republic Of

## Abstract

**Introduction:**

Depression often coexists with cognitive impairment and may act as both a risk factor and a symptom of cognitive decline. Functional loss itself can also trigger depressive symptoms. Prior research shows associations between depressive symptoms and activities of daily living (ADL), though results differ due to varied populations and settings. Since cognition impacts daily functioning, this study examines the independent role of depression and cognition in functional dependence among older adults with cognitive complaints.

**Objectives:**

To investigate whether depressive symptoms predict dependence in basic ADL and instrumental ADL (I-ADL) independently of global cognition. Because >50% scored zero on ADL (independence), analyses excluding these participants were conducted to address ceiling effects.

**Methods:**

Retrospective data from patients with cognitive complaints who completed Mini-Mental State Examination (MMSE), Geriatric Depression Scale (GDS), and ADL/I-ADL assessments were analyzed. Correlation and regression analyses were performed on the full sample and excluding ADL=0 cases (Table 1). Group comparisons examined differences between those with and without basic ADL dependence. Figure 1 shows scatter plots.

**Results:**

MMSE and GDS correlated significantly with ADL and I-ADL, but not with each other. In the full sample (N=69), both depressive symptoms (β=0.333, p<0.001) and cognition (β=–0.355, p=0.002) predicted basic ADL dependence (R²=0.312). However, model assumptions were violated due to many ADL=0 cases. After excluding these (N=32), only depressive symptoms predicted ADL dependence (β=0.486, p=0.004; R²=0.265). For I-ADL (N=69), cognition alone predicted dependence (β=–1.307, p<0.001; R²=0.387). Participants with ADL=0 had higher MMSE scores (19.4±5.0 vs. 14.7±5.4, p=0.001), lower GDS scores (16.4±6.4 vs. 20.3±6.8, p=0.018), and lower I-ADL scores (7.1±4.7 vs. 25.4±10.8, p<0.001) than those with ADL dependence. Age did not differ significantly (p=0.526).

**Table 1.** Multivariate regression predicting dependence in ADL and I-ADL Table 1. Long description.

Model	Dependent variable	Predictor	β	p-value	R²
Full sample (N=69)	ADL	GDS	0.333	<0.001	0.312
		MMSE	–0.355	0.002	
Excluding ADL=0 (N=32)	ADL	GDS	0.486	0.004	0.265
Full sample (N=69)	I-ADL	MMSE	–1.307	<0.001	0.387

**Image 1:**
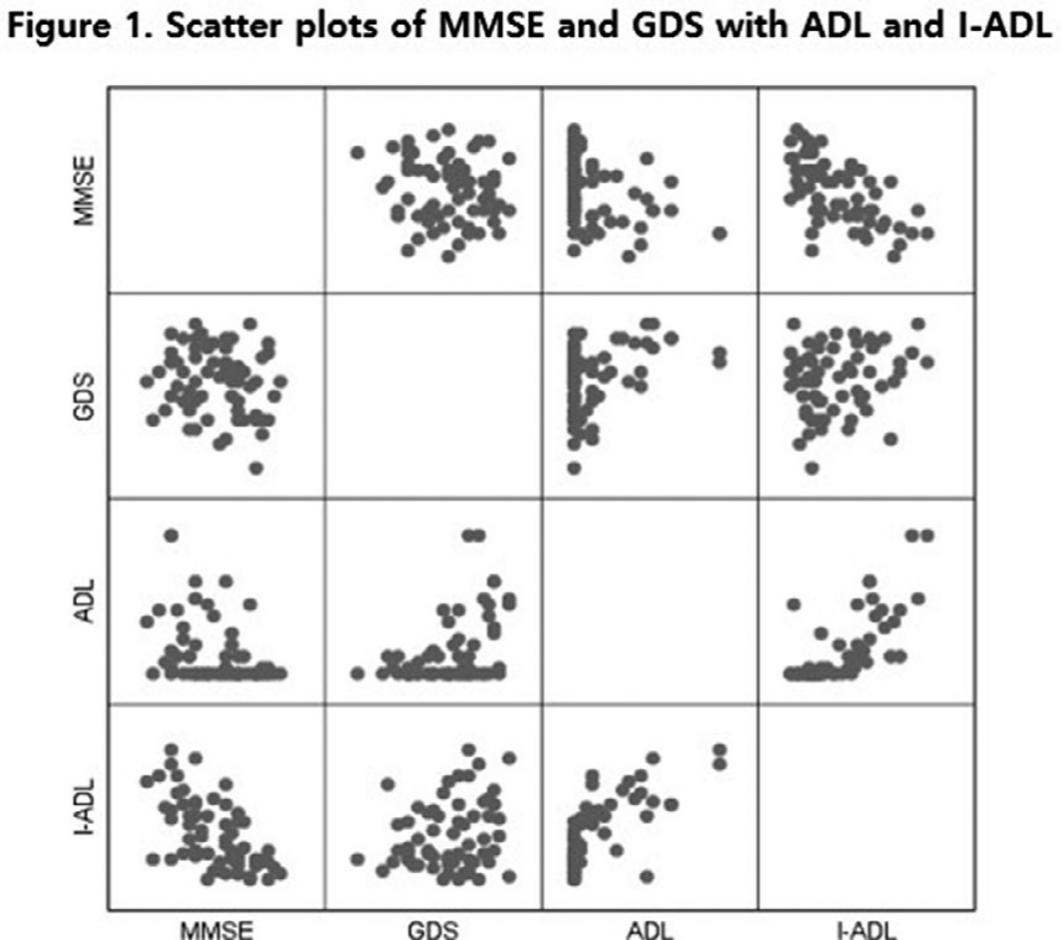
Image 1: Long description.

**Conclusions:**

Depressive symptoms independently predict basic ADL impairment in those with functional decline, suggesting mood disturbances play a key role in basic daily functioning loss. Cognitive impairment predicts instrumental ADL dependence but not basic ADL in this subgroup. Findings highlight the need for assessment and treatment of depressive symptoms to maintain daily functioning in older adults with cognitive complaints.

**Disclosure of Interest:**

None Declared

## Long descriptions

### Table 1. Long description

The table consists of six columns: Model, Dependent variable, Predictor, beta, p-value, and R super 2.

* The first model, Full sample with N equals 69, predicts A D L. It includes two predictors: G D S with a beta of 0.333 and p-value less than 0.001; and M M S E with a beta of minus 0.355 and p-value of 0.002. The R super 2 for this model is 0.312.

* The second model, Excluding A D L equals 0 with N equals 32, predicts A D L using the predictor G D S. It shows a beta of 0.486 and a p-value of 0.004, with an R super 2 of 0.265.

* The third model, Full sample with N equals 69, predicts I A D L using the predictor M M S E. It shows a beta of minus 1.307 and a p-value less than 0.001, with an R super 2 of 0.387.

Navigate back to Table 1..

### Image 1: Long description

The matrix displays 12 scatter plots across 16 panels. The diagonal panels from top-left to bottom-right are empty. The variables are M M S E, G D S, A D L, and I-A D L.

* Top row (Y-axis M M S E): The second panel (X-axis G D S) shows a scattered distribution with a slight negative trend. The third panel (X-axis A D L) shows data clustered at low A D L values. The fourth panel (X-axis I-A D L) shows a moderate negative correlation.

* Second row (Y-axis G D S): The first panel (X-axis M M S E) mirrors the top row with a slight negative trend. The third panel (X-axis A D L) shows a vertical cluster at low A D L values. The fourth panel (X-axis I-A D L) shows a weak negative correlation.

* Third row (Y-axis A D L): The first panel (X-axis M M S E) shows most data points at the bottom of the Y-axis. The second panel (X-axis G D S) shows a similar bottom-heavy distribution. The fourth panel (X-axis I-A D L) shows a strong positive linear correlation.

* Bottom row (Y-axis I-A D L): The first panel (X-axis M M S E) shows a moderate negative correlation. The second panel (X-axis G D S) shows a weak negative correlation. The third panel (X-axis A D L) shows a strong positive linear correlation.

Navigate back to Image 1:.

